# Application of Marte Meo® counselling with people with behavioural variant frontotemporal dementia and their primary carers (AMEO-FTD) – a non-randomized mixed-method feasibility study

**DOI:** 10.1186/s40814-020-0551-1

**Published:** 2020-02-26

**Authors:** Martin Berwig, Claudia Dinand, Ursula Becker, Margareta Halek

**Affiliations:** 1 German Centre for Neurodegenerative Diseases (DZNE), Witten, Stockumer Straße 12, 58453 Witten, Germany; 2 School of Nursing Science, Faculty of Health, Witten/Herdecke University, Witten, Germany; 3 Clinic for Cognitive Neurology [Day Care Unit], Medical Faculty, University of Leipzig, Leipzig, Germany; 4 Psychotherapy Practice and Marte Meo® Training Institute, Alfter, Germany

**Keywords:** Behavioral variant frontotemporal dementia, Video feedback, Marte Meo®, Counselling, Communication training, Dyadic intervention, Interaction, Relationship quality

## Abstract

**Background:**

One of the core symptoms of behavioural variant frontotemporal dementia (bvFTD) is the early loss of social cognitive abilities, which has a deteriorating impact on everyday interaction and the quality of dyadic relationships. Marte Meo® (MM) counselling is a video-based intervention that aims to maintain or improve the quality of dyadic relationships. This non-randomized mixed-method study aimed to evaluate the feasibility of the intervention in practice with primary carers of persons with bvFTD as well as the feasibility of a future confirmatory trial.

**Methods:**

A pilot effect study with a quasi-experimental, one-group, pre-post design and double pre-measurement was conducted. Data were collected at three time points (t0, t1 after 2 weeks, and t2 after 6 weeks) using videography and several measurement instruments. Between t1 and t2, each primary carer received five MM counselling sessions. The outcomes included positive and negative affect, behavioural and psychological symptoms in dementia (BPSD), the interpersonal abilities of the person with dementia, the sensitivity and distress of the primary carers due to BPSD, the manageability of BPSD, the personal goal attainment by means of MM counselling, and the quality of the dyadic relationships. The pilot process evaluation focused on the primary carers’ and the interventionist’s perceived benefits and perceptions of the intervention process using questionnaires and interviews.

**Results:**

Five dyads were enrolled. Regarding the feasibility of the intervention, MM counselling seems to be appropriate and useful for the target group. Although the recruitment of persons with reliable bvFTD diagnoses was very time consuming and complex, the intervention was well accepted by the dyads, and regarding goal attainment, all carers benefited as much or even more than they expected. The study also showed that the benefits of MM counselling depend on whether the primary carer has accepted his/her relative’s dementia. Regarding the feasibility of a future confirmatory trial, certain outcomes, particularly positive affect, distress due to BPSD, and the quality of the dyadic relationship, seem to be appropriate for describing possible effects.

**Conclusion:**

Overall, the intervention seems feasible for this target group. A future confirmatory trial should be planned as a multicentre pilot trial with an extension option.

**Trial registration:**

DRKS00014377. Registered retrospectively on April 11, 2018.

**Electronic supplementary material:**

The online version of this article (10.1186/s40814-020-0551-1) contains supplementary material, which is available to authorized users.

## Background

Frontotemporal dementia belongs to a group of neurodegenerative changes caused by various protein deposits in the region of the frontal and temporal lobes [1, 2]. These changes are referred to as frontotemporal lobar degeneration (FTLD). Depending on the locations of the deposits, they can have different effects on language ability, emotions and social behaviour, which are a source of stress, burden and decreased quality of life (QoL) for people with FTLD and their carers [3].

The disease is generally rapidly progressing. It can equally affect men and women [4] from the age of 30 but is especially frequent beginning in the sixth decade of life [5]. Life expectancy after diagnosis is short and ranges from 1.3 to 6.5 years [6]. Most of them are living at home, cared for by a relative [7].

Data on the prevalence of FTLD vary internationally, showing a prevalence of between 1 and 461/100,000 persons [8] and 2–31/100,000 persons in the 45–64 age group [4]. In Germany, the total number of cases is estimated at approximately 33,000 [9]. A general distinction is made between behavioral variant frontotemporal dementia (bvFTD) and two language-specific forms: semantic dementia and primary progressive aphasia. Other less common subtypes may also be associated with motor neuron diseases or Parkinson’s disease [2]. BvFTD represents the most common subtype of FTLD [2] and is the subject of this study.

### Behavioral variant frontotemporal dementia and social cognition

According to recent diagnostic criteria [10], a progressive change in personality and social interpersonal behaviors is paramount to bvFTD. Roca et al [11] found that the specific ability to integrate (social) context information and subsequently the ability to communicate [12] is already impaired at a very early stage of the disease [13–15]. Specifically, such ability refers to, e.g. the ability to recognize facial expressions, to empathize or to mentalize, viz. the ability to imagine other people’s feelings, beliefs, opinions and desires or even to have a mental concept of having another person’s own intentions, feelings and opinions. All these abilities are summarized under the term “social cognition” [16]. Impaired social cognition and associated impaired social functioning as a first and core symptom is also a developmental problem observed with autism but not primarily with Alzheimer’s disease or Down’s syndrome [17]. Thus, relatives of persons with bvFTD often report the affected persons’ increasing coldness and lack of empathy for relatives, friends and family. The affected persons themselves often do not notice that they react differently to their environments than before and often do not have insight into their illness [10, 18]. Due to severely impaired emotional connections and interactions (quality of relationship), family carers of people with bvFTD are also more burdened by providing care and support and coping with everyday life than carers of people with Alzheimer’s disease [3, 7]. In addition, there is a lack of adequate support systems for the early phase of life [19–22]. Therefore, planning and delivery of services should be adapted for people with FTLD syndromes [23].

For FTLD conditions, psychosocial interventions are still considered first-line interventions [24]. In particular, interventions for the education and support of the primary carers as well as dyadic interventions are currently the most important components of clinical management and the most recommended interventions [25]. However, only a few larger scaled confirmatory trials have been conducted in this area [26], and therefore, little evidence of the effects and benefits of psychosocial interventions is available. This lack of evidence shows that there is still a great need either to develop new psychosocial interventions for each subtype of FTLD or to transfer interventions designed for other patient groups especially for bvFTD. Against this backdrop, in this study, we applied a counselling method called Marte Meo® (MM) with people with bvFTD and their primary carers. Since staying in contact and maintaining good-quality relationships are the key problems in cases of bvFTD and since most people with bvFTD live at home and are cared for by their primary carers [27], MM might be a promising counselling approach in this target group.

### Marte Meo® counselling as a dyadic intervention

**Fig. 1:**
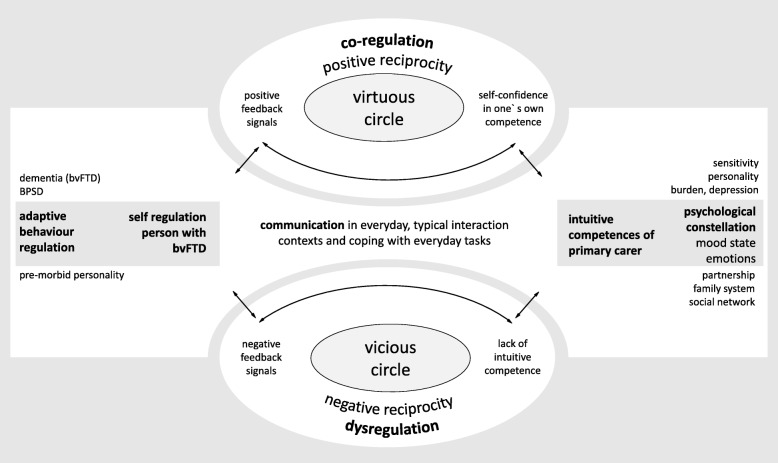
Model of communication between a person with bvFTD and his/her primary carer (modified from [30, 31]). Legend: bvFTD: behavioural variant frontotemporal dementia; BPSD = Behavioural and Psychological Symptoms of Dementia

Marte Meo® is a counselling method, which was originally developed to support parents of children with autism [28, 29]. The term “Marte Meo” is derived from the Latin “mars martis”, a term used in mythology that means “on one’s own strength” [29]. The method uses video feedback as an indirect intervention to strengthen the intuitive competence of a family carer to communicate with a person entrusted to his/her care. The focus of MM counselling is the analysis and (co-)design of dialogues that involve communication and interaction processes. Figure 1 shows the dynamics and the processes of communication between a person with bvFTD and his/her primary carer and their central moderating factors.

In this context, “dynamic” refers to the self-organized direction of development towards positive or negative valences by means of communication. Although not shown in Fig. 1, a middle range of “normal” or “neutral” valence also exists, which is also widespread in everyday communication. The aim of Fig. 1 is to illustrate the self-reinforcing process of the orientation of communication with the co-regulating and moderating framework conditions. The information that is important for video feedback is derived from the observation of “virtuous circles”. The purpose is to search for successful moments, and the systematic observation of these successful moments can also help transform “vicious circles” into a positive dynamic, which is ultimately reflected in a better-quality dyadic relationship [30]. According to MM, these ultrashort moments (< 1 s) are essential, universal elements occurring in every human dialogue, although they have to be adapted to different life situations, e.g. to dementia care [28]. These moments are called function-supporting elements (FSEs) (see Table 1) [28] and are identified by the use of video feedback.

**Table 1:** Function-supporting elements according to Marte Meo® [28]

Nr.	Function-supporting element (FSE)
1.	Prepare for a good beginning and a positive atmosphere through tone and eye contact
2.	Locate, confirm and follow the person’s focus
3.	State what is happening, what is going to happen, and what is experienced
4.	Reinforce coping ability by providing help to start and end an activity
5.	Help the person with dementia to be in rhythm in the dialogue by waiting for an answer or supporting the persons initiatives
6.	Help or support the person with dementia to respond to new people or situations in the setting
7.	Pay attention to physical contact
8.	Lead in a positive way

Providing this information in the counselling process makes the interaction visible and comprehensible, increases the primary carer’s awareness of FSEs and offers opportunities for the carer to shift from perceiving his/her own actions as randomly successful moments to recognizing them as helpful tools in establishing relationships. The use of video and the possibility to replay segments, for instance, in slow motion, is uniquely useful for making these ultrashort communication elements visible and thereby increasing awareness [30].

MM counselling is a method adopted since the beginning of the new millennium to support staff recognise and improve their own communication and interaction when working with people with dementia [32]. To the best of our knowledge, some evidence based on a qualitative exploratory intervention study on MM counselling for people with Alzheimer’s disease in nursing homes showed an increase in successful dyadic interactions [28]. Additionally, a feasibility study on video feedback at home reported some positive outcomes in enhancing carers’ communication skills in terms of insights, acceptance, coping and self-confidence [33]. In this feasibility study, MM counselling was applied and systematically evaluated in people with bvFTD and their primary carers for the first time.

## Methods

A non-randomized mixed-method feasibility study was undertaken following the methodological framework for the development and evaluation of complex interventions stipulated by the Medical Research Council (MRC) [34]. The evaluation of this feasibility study took place on two levels: (a) a pilot effect study and (b) a pilot process evaluation.

### Pilot effect study

#### Aims and objectives

For the pilot effect study, the following two research questions were addressed to provide essential preparatory work to assess the feasibility of a future confirmatory trial:

- What are suitable outcomes and associated recording instruments for evaluating the effects of the intervention?
- Are there descriptively shown effects in favour of the intervention and are these effects clinically significant?

#### Design/data collection

**Fig. 2:**
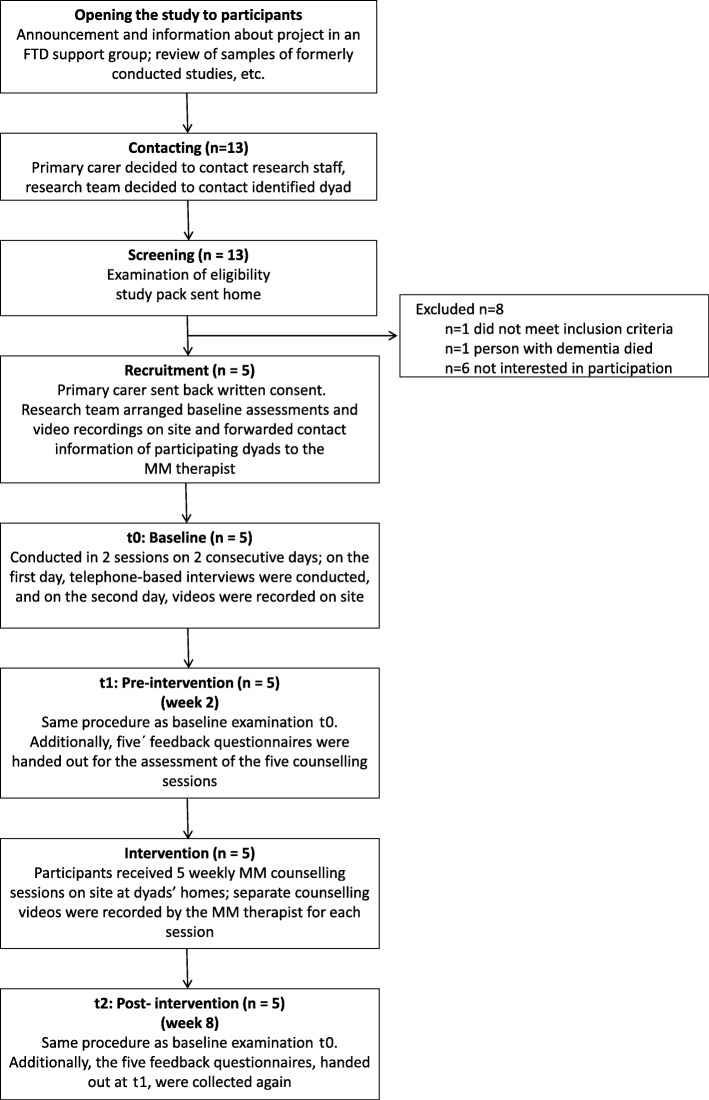
CONSORT flow diagram of the AMEO-FTD study. Legend: MM: Marte Meo®

The pilot effect study used a quasi-experimental, one-arm, pre-post design with double pre-measurement and an embedded qualitative change evaluation [35] with three examination points: t0 (baseline), t1 (pre-intervention) and t2 (post-intervention). During the 2-week control period between t0 and t1, none of the participating carers received the intervention; they only received the intervention during the 5-week intervention period between t1 and t2 (see Fig. 2).

The data were collected using quantitative interviews based on standardized questionnaires and videography at all three examination points (t0, t1 and t2) in two sessions on two consecutive days (see Fig. 2 and Table 2). On the first day, interviews were conducted by phone, and on the following day, separate research videos (in addition to counselling videos) of dyadic interactions between the persons with bvFTD and their primary carers during a daily activity were recorded. We used a fixed camera on site, which complemented the videos with ethnographic contextual information. For the comparability of the observation data, the same activity was chosen for all participants. The (lunch) mealtime was chosen, as it seemed to be a universal activity with interaction potential.

**Table 2:** Core set of instruments used

Outcome/domain	Instrument	Data source	t0	t1	t2^a^
Pilot effect study (with an embedded qualitative change evaluation)^b^					
*bvFTD*					
▪ Relationship ability	MM instrument [36]	Evaluations of videos of dyadic interactions by researchers	●	●	●
▪ Affect	QUALIDEM positive and negative affect subscales [37]	Proxy rating by primary carer	●	●	●
▪ Behavioural and psychological symptoms in dementia (BPSD)	NPI [38]	Proxy rating by primary carer	●	●	●
*Primary carer*					
▪ Sensitivity	SI [39]	Assessment of videos of dyadic interactions by researchers	●	●	●
▪ Stress experience due to BPSD	NPI-D [38]	Self-rating	●	●	●
▪ Manageability of BPSD	NPI-M [40]	Self-rating	●	●	●
▪ Goal attainment by intervention	GAS [41]	Self-rating		●	●
*Dyad*					
▪ Quality of relationship	QCPR scale [42, 43]	Assessment by primary carer	●	●	●
▪ Social Interaction^b^	Videography of dyadic interaction in everyday life [44]	Videos, video transcripts and context information of researchers	●	●	●
Pilot process evaluation					
*Intervention process*					
▪ Benefits and perceptions of counselling	Quantitative interviews with 35 standardized single items	Primary carer			●
▪ Benefits and perceptions of counselling	Quantitative feedback questionnaire with standardized 11 single items	Primary carer			
▪ Promoting and inhibiting factors, appropriateness for the target group, doses and frequency, quality of counselling, etc.	Guided telephone interviews	Marte Meo® Therapist			●
*Study process*					
▪ Recruitment rate	Recruitment documentation	Researcher			
▪ Recruitment, data collection, confirmation of bvFTD diagnosis	Field notes, documentation and memory protocols, reflections and observations on site	Researcher			

^a^If there were data collection activities between t0, t1 and t2, the time points are interconnected with a solid line

^b^Methodological details and the results of the embedded qualitative evaluation will be reported elsewhere

*bvFTD* behavioural variant frontotemporal dementia, *MM* Marte Meo®, *NPI* Neuropsychiatric Inventory, *QUALIDEM* proper name, *SI* Sensitivity index, *NPI-D* NPI Caregiver Distress Scale, *NPI-M* NPI Caregiver Manageability Scale, *GAS* Goal Attainment Scale, *QCPR* Quality of Carer-Patient Relationship (QCPR) scale

#### Sample

A maximum of five dyads were possible to be enrolled, because internal budget for this study was restricted. However, this number seemed to us sufficient for the purpose of assessing basic aspects of feasibility with special regard to assessment of feasibility of the intervention.

Dyads consisting of a person with bvFTD and his/her primary carer were eligible to participate if they met the following inclusion criteria: the person to be cared for had a confirmed (by a medical specialist) diagnosis of bvFTD, the severity of bvFTD had been determined with the Frontotemporal Dementia Rating Scale (FRS) [45]), the person caring for the person with bvFTD was the primary carer and the dyad members were living in Central Germany at the time of the study. Moreover, the following exclusion criteria were applied: insufficient knowledge of German, parallel participation of the person with bvFTD or the primary carer in another intervention study, presence of serious psychiatric illness of the primary carer or presence of a different form of FTLD other than bvFTD in the person with dementia.

Dyads were recruited with an announcement and information about the project in a support group for primary carers of people with FTLD and through a review of samples of formerly conducted studies. Additionally, one participating dyad was recruited by using personal contacts of the research team.

#### Intervention

MM counselling was provided by a certified MM therapist who was experienced in using MM counselling with people with dementia and their primary carers. The MM therapist provided counselling individually and face to face. The counselling sessions took place at the home of each dyad in weekly intervals over a period of 5 weeks. Each session lasted until the session content was complete. Once the objectives and expectations of the primary carers regarding the MM counselling had been established by the MM therapist, the same therapist recorded short video sequences of daily dyadic interactions between the person with bvFTD and his/her primary carer in open situations (e.g. game situations) or situations requiring structure (e.g. mealtime situations). These recordings were analysed by the MM therapist, and selected clips were used as video feedback to demonstrate successful dyadic interactions to the primary carers based on the FSEs (see Table 1). Additional information was provided on how these FSEs affected the persons with bvFTD. As instruments of quality assurance, the MM therapist also recorded the MM counselling on video and was occasionally supervised by an MM colleague. At the end of each counselling process, the MM therapist independently created a compilation of all the counselling videos of each dyad and the central counselling contents and provided it to the dyad.

#### Outcomes/instruments

Several outcomes and corresponding instruments were determined and sorted by the target group (see Table 2).

##### Persons with bvFTD

1. *Relationship ability*: The video sequences recorded at t0, t1 and t2 provided data to assess the interpersonal relationship abilities of the person with bvFTD by the MM instrument on four domains [46]: the frequency of inter-intentionality, extent of inter-affectivity (scored from 1 to 5), time of shared attention focus (sec) and frequency of communication circles concerning one topic. For each domain a separate score is generated; a total score is not used. The MM instrument was originally developed to assess the state of development of children’s interpersonal abilities [36]. For this study, we adapted the instrument for use with people with dementia and in particular to assess the remaining relationship abilities of the person with bvFTD. Two student assistants in nursing science rated videos from t0, t1 and t2 to determine interrater reliability [47].
2. *Affect*: Positive versus negative affect were assessed with the positive affect (scores from 0 to 36) and negative affect (scores from 0 to 18) dimensions of the German version of the QUALIDEM [37]. Higher scores indicate a higher extent of affect. The assessment tool has good reliability and is suitable for measuring QoL in people with mild to severe dementia.
3. *Behavioural and psychological symptoms of dementia (BPSD)*: The Neuropsychiatric Inventory-Nursing (NPI) [38], which is a common and validated instrument for detecting BPSD in elderly people with dementia [48], includes items for delusions, hallucinations, agitation, depression, anxiety, euphoria, apathy, disinhibition, irritability, aberrant motor behaviour, night-time disturbances, and eating abnormalities. For each of the 12 items, the presence, frequency, and severity can be assessed. The severity and frequency of each symptom are scored on the basis of the carer’s responses to structured questions. The score for each symptom is obtained by multiplying the severity (1–3) by the frequency (1–4). The summed symptom scores give the total NPI score, which ranges from 0 to 144. Higher values correspond to more frequent and severe behaviour. In our study, we used a validated German version of the NPI [40].

##### Primary carers

(4) *Sensitivity of primary carers*: There is currently only one instrument available to measure sensitivity in nursing interactions: the sensitivity index (SI) [39]. The SI is an observational instrument with 15 items used to assess the extent of sensitivity of carers in three skills areas (domains): making offerings (5 items), physical expression (6 items) and use of language (4 items). The items are scored from 0 = “not observable” to 4 = “consistently present”, with higher scores indicating more sensitivity. Since the psychometric properties of this instrument are not yet published, no sum scores (domains or total scores) were used. The interrater reliability [47] was determined by two trained nursing science and psychology student assistants who rated the videos from t0, t1 to t2.
(5) *Stress experience due to BPSD*: The primary carers assessed their stress experience due to the BPSD of the person with bvFTD with the NPI Caregiver Distress Scale (NPI-D). Stress experience due to each of the 12 symptoms included in the NPI is rated on a 5-point Likert scale from 1 to 5, and the corresponding total NPI-D score ranges from 0 to 60. Higher values indicate greater stress experience due to BSPD.
(6) *Manageability of BPSD*: The manageability of BPSD was assessed by the primary carers with the NPI Caregiver Manageability Scale (NPI-M) [40]. The manageability of each of the 12 symptoms included in the NPI is rated on a 5-point Likert scale from 0 to 4, and the total NPI-D score ranges from 0 to 48. Higher values indicate a better manageability of BSPD.
(7) *Goal attainment*: To assess the extent to which the primary carer of the person with bvFTD was able to reach his/her own goals through MM counselling, Schäfer’s Goal Attainment Scale (GAS) [41] was applied. The instrument first requires the respondent to define one or more specific and tangible objectives. To assess the achievement of these indicators, indicators are described for each goal (see Additional file 1: Table S1).

##### Dyads

(8) *Quality of the relationships between persons with bvFTD and their primary carers*: There is currently no German instrument to capture the quality of a relationship. However, a well-validated original version of the instrument in Flemish and English exists [42, 43]: the Quality of Carer-Patient Relationship (QCPR) scale. The scale consists of 14 items, which measures the quality of the relationship on two dimensions: criticism or lack of criticism (7 items) and warmth and affection (8 items). The responses are scored on a 5-point Likert scale, ranging from “disagree” to “totally agree”. We used only the total score, which ranges from 0 to 70. In preparation for the present study, the QCPR was translated into German following the guidelines for the transcultural adaptation of self-assessment instruments by Beaton and colleagues [49] (see Additional file 2: Table S2 for the German version of the instrument).
(9) *Social interaction.* Videography based on focused ethnography according to Knoblauch [44] was used as a qualitative approach to identify patterns of social interaction as well as patterns of changes in the dyadic interactions between people with bvFTD and their primary carers in their everyday life routines. Therefore, all research videos and related ethnographic contextual information (see above) were used for interpretation (see Table 2). The methodology used to evaluate the videographic data (video interaction analysis, VIA [44]) and the results of this evaluation will be published elsewhere.

#### Pilot effect analysis

The interrater reliability for each item of the MM instrument and the SI was determined by Krippendorf’s alpha. To reduce reactivity effects and to improve the quality of the raw data, only the middle 4-min sequence of each of the recorded research videos (with durations of 10–27 min) was assessed with the MM instrument and the SI. These video sequences were blinded before data assessment. The pilot effect analysis for the MM instrument and the SI was conducted on the item level, whereby only items with alphas ≥ 0.667 were analysed [47].

Overall, the effect analysis was not focused on inferential statistical generalizations of the observed effects on a population but with the assessment of the effect direction as well as the clinical significance of the observed effects within the sample. If no critical differences exist between the used instruments, the absolute effect size can be determined by convention or by comparison with the theoretical scale width [50]. Therefore, percentage effect sizes were calculated by converting the absolute differences or changes to a standard scale of 1 to 100 (C-values). A value of 7.8 is a typical percentage effect for empirical social research [50]. In the verbal description of value differences, we were guided by a rough classification from Lind [50]: effect > 10% of the scale width = “very significant” or “very clear”; effect > 5% of the scale width = “significant” or “clear”. Moreover, the ratings of the GAS indicators for personal goal attainment were descriptively analysed and outlined (see Additional file 1: Table S1).

### Pilot process evaluation

#### Aims and objectives

For the pilot process evaluation, the following three sets of research questions were addressed to provide essential preparatory work to assess the feasibility of the intervention in practice:

- What are expectations and experiences of the primary carers, the MM therapist and the scientific staff regarding the intervention? What are promoting and inhibiting factors of dyadic interactions?
- Was the intervention carried out as intended? Is the intervention suitable for people with bvFTD? Are the contents appropriate? Are there potential modification approaches? What is a suitable intensity (dose) of the intervention?
- How can the recruitment process be initiated with the target group? What motivates the target group to participate in counselling and the study? What is the willingness of the actors in the health care system to participate in the acquisition of participants? Are the inclusion and exclusion criteria suitable for a future confirmatory trial?

#### Design/data collection

For the pilot process evaluation, quantitative interviews based on standardized single items were conducted with the primary carers via telephone at the t2 measurement point (please refer to the “pilot effect evaluation” section), and quantitative feedback questionnaires based on standardized single items to be completed by the primary carers after the end of each counselling session were distributed on site on the second day of the t1 measurement. Furthermore, after the end of each counselling process, a qualitative interview with the MM therapist was conducted via telephone.

#### Sample

In addition to the participating dyads (please refer to the “pilot effect study” section), the MM therapist and the researcher, who was responsible for data collection and recruitment, were included in the pilot process evaluation.

#### Domains/instruments

The pilot process evaluation focused on the interventions as well as the study process (see Table 2).

##### Intervention process

(10) *Benefits and perception of the counselling*: The

**Fig. 3:**
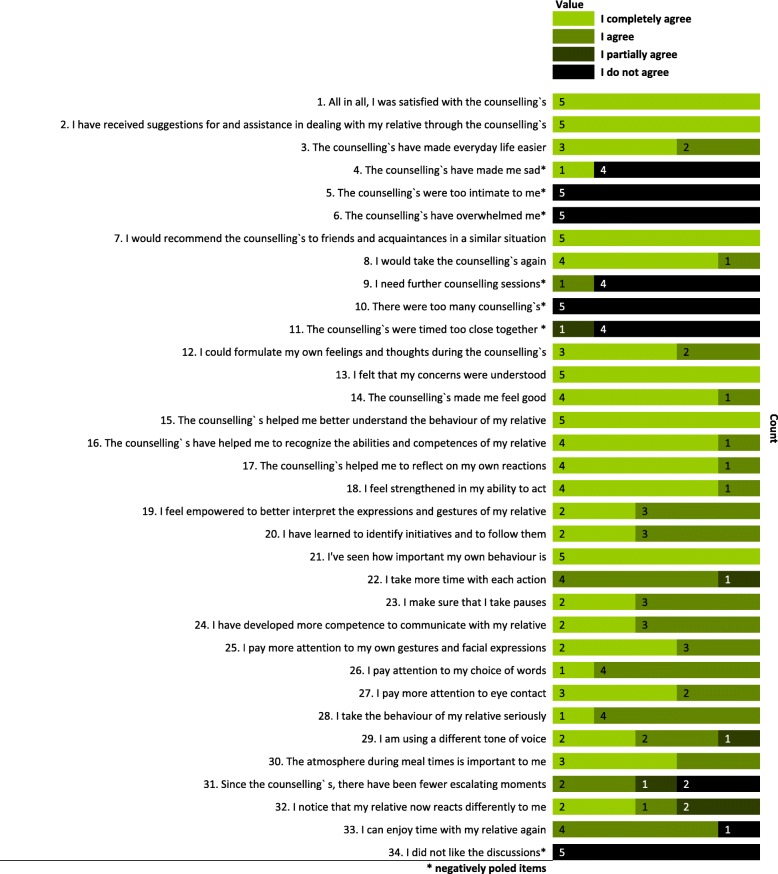
Results of pilot process evaluation for quantitative interviews

**Fig. 4:**
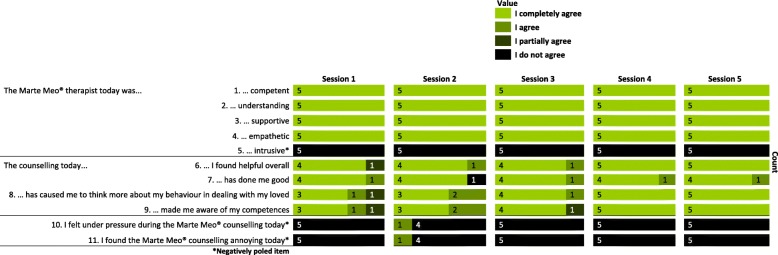
Results of the pilot process evaluation for the quantitative feedback questionnaires
(11) carers’ personal benefits and perceptions of the intervention were assessed with 35 single items (27 positively and seven negatively poled items) in the quantitative interviews at t2 and 11 single items (eight positively and three negatively poled items) in the quantitative feedback questionnaire after each MM counselling session. The single items were statements reflecting the subjective process of MM counselling. There were four response options: “I completely agree”, “I agree”, “I partially agree” and “I do not agree” (see Figs. 3 and 4).
(12) *Promoting and inhibiting factors, appropriateness for the target group, doses and frequency, quality of counselling, etc*.: To address the perceptions of the intervention regarding themes such as the promoting and inhibiting factors for the intervention process, the appropriateness of the intervention for the target group, the doses and frequency of MM counselling sessions and the quality of the conducted counselling, individual interviews following a qualitative interview guide were conducted with the MM therapist. The interviews were audiotaped and transcribed verbatim without notations.

##### Study process

(13) *Recruitment rate*: The recruitment rate was determined on the basis of the recruitment documentation.
(14) *Recruitment, data collection, confirmation of bvFTD diagnosis*: Regarding the recruitment and data collection processes, field notes, documentation and memory protocols and observations of the researchers on site were collected during the study.

#### Pilot process analysis

Concerning the pilot process analysis, the absolute frequency for each response category of the single items from the interviews with the primary carers and the feedback questionnaires was descriptively analysed. Furthermore, the researchers’ documentation of the study was superficial, structured based on the content of the data and focused on the recruitment and data collection process. The transcribed interviews with the MM therapist were analysed by means of qualitative structural content analysis according to Kuckartz [51] using MAXQDA® 18.1 software for qualitative data analysis. The codes were formed deductively based on the interview guideline and inductively from the data material. Fidelity was assessed by evaluating the qualitative data from an interview with the MM therapist after the end of each MM counselling process with one dyad.

### Ethics

Before enrolment, the primary carers gave informed consent to participate after receiving written information in the mail and verbal information during phone calls prior to the data collection. A proxy consent was obtained for all persons with bvFTD by the legal representative or through the authorization of a precautionary power of attorney related to health based on the prior or presumed will to participate in the study. Ongoing consent [52] was respected during data collection at all times.

The Ethics Committee of the German Society for Nursing Sciences (Deutsche Gesellschaft für Pflegewissenschaft, DGP) approved the study before the enrolment of participants.

## Results

Altogether, five dyads were enrolled in the study over the course of 10 months (from July 2016 until April 2017). The baseline characteristics of the dyads are presented in Table 3. The mean age of the persons with bvFTD was 60 years, and the mean age of the primary carers was 62 years. The educational level of the primary carers was high, with a mean of 14 years of education [53]. Of the 5 primary carers, 4 were wives; the carer with a non-marital relationship to the person with bvFTD was a mother caring for her daughter. Four of the five persons with bvFTD were already in a severe to very severe stage of the disease, as determined by the FRS [45].

**Table 3:** Characteristics of the dyads (a person with bvFTD and his/her primary carer)

Variable	*M ± SD* or absolute frequencies *N* = 5
Primary carer	
Age (year)	62.20 ± 12.34
Sex, male:female	0:5
Education (years)	14.20 ± 2.56
Relation to person with dementia, spouse:parent	4:1
Duration of care (months)	64.60 ± 62.26
Occupational status, retired:part-time employed:full-time employed	3:2:0
Household income [€]: < 1000:1000–1500:> 1500	1:1:3
Joint household with person with bvFTD, yes:no	4:1
Nursing support, yes:no	1:4
Day care, yes:no	2:3
Person with bvFTD	
Age (years)	60.00 ± 16.94
Sex, male:female	4:1
bvFTD confirmed by research staff, yes:no	5:0
FRS stages, very mild:mild:moderate:severe:very severe:profound	0:0:1:2:2:0
Care level, I:II:III	2:2:1

*M* mean, *SD* standard deviation, *bvFTD* behavioural variant frontotemporal dementia, *FRS* Frontotemporal Dementia Rating Scale

### Pilot effect analysis

The results of the pilot effect analysis are presented in Table 4. From all the items of the two instruments measuring the interpersonal abilities of the person with bvFTD (the MM instrument) and the sensitivity of the primary carer (the SI), only two items from the MM instrument could be analysed because of insufficient reliability: inter-intentionality and shared attention focus (joint eye contact). Moreover, the QCPR scale was administered only to the primary carers because the participating persons with bvFTD were not able to provide this assessment due to the severity of disease.

**Table 4:** Results of the pilot effect study

Variable	*N* = 5	Post-controlchange from t0 to t1	Post-interventionchange from t1 to t2	Effect direction^a^	Scale width	Absolute effect	Percentage effect
		*M*_*t1-t0*_ *± SD*	*M*_*t2-t1*_ *± SD*				
Person with bvFTD							
Affect (QUALIDEM), CR							
Positive affect		0.20 ± 3.70	5.80 ± 4.92	+	36	5.6	15.6**
Negative affect		0.40 ± 2.30	−0.40 ± 1.82	−	18	0.8	4.4
BPSD (NPI), CR		−7.20 ± 9.26	−11.80 ± 13.42	−	144	4.6	3.2
Interpersonal ability (MM instrument), RR							
Inter-intentionality [FREQ]		6.30 ± 6.49	−1.80 ± 12.52	(−)	∞	8.1	n. c.
Shared attention focus							
Joint eye contact [s]		−6.34 ± 8.83	−3.57 ± 15.97	(−)	∞	3.2	n. c.
Jointed object reference [s]		n. e.	n. e.	n. e.	∞	n. e.	n. e.
Mutual affect tuning		n. e.	n. e.	n. e.	5	n. e.	n. e.
Turn-taking and passing a…^b^ [FREQ]		n. e.	n. e.	n. e.	∞	n. e.	n. e.
Primary carer							
Sensitivity (SI), RR							
SI items 1–15		n. e.	n. e.	n. e.	45	n. e.	n. e.
Distress due to BPSD (NPI-D), SR		0.40 ± 4.39	−9.00 ± 7.07	−	60	9.4	15.7**
Manageability of BPSD (NPI-M), SR		−0.60 ± 4.45	−2.00 ± 3.16	(−)	48	1.4	2.9
Dyad							
Quality of relationship (QCPR scale), CR		−2.60 ± 2.51	3.60 ± 7.27	+	70	6.2	8.9*

^a^ “+” | *M*_t2-t1_-*M*_t1-t0_ > 0 and “−” | *M*_t2-t1_-*M*_t1-t0_ < 0; if the effect direction was not in favour of the intervention period, the sign is put in brackets

^b^ … communication circle

t0 = baseline, t1 = pre-intervention (after 2 weeks), t2 = post-intervention (after 5 weeks), *N* number, *M* mean, *SD* standard deviation, *bvFTD* behavioural variant frontotemporal dementia, *QUALIDEM* proper name, *CR* carer rating, *BPSD* behavioural and psychological symptoms in dementia, *MM* Marte Meo®, *NPI* Neuropsychiatric Inventory, *NPI-D* NPI Caregiver Distress Scale, *SR* self-rating, *NPI-M* NPI Caregiver Manageability Scale, *RR* researcher rating, *n. c.* not calculable, *FREQ* frequency, *n. e.* not evaluable, *sec* seconds, *SI* sensitivity index, *QCPR* Quality of Carer-Patient Relationship; * clinically significant (percentage effect > 5%), ** clinically very significant (percentage effect > 10%)

In five of the following eight evaluable outcomes or instruments, the effect direction was in favour of the intervention period: positive and negative affect (QUALIDEM), BPSD (NPI), stress experience of the primary carer due to BPSD (NPI-D) and quality of the dyadic relationship (QCPR scale). The mean percentage effect was 8.45, whereby the percentage effect sizes (C-values) for the QUALIDEM subscales of positive affect and negative affect and the NPI-D could be described as clinically very significant and those of the QCPR scale could be described as clinically significant. The predefined goals levels of the GAS (e.g. maintaining stress or a positive mood during an interaction with the person with bvFTD, remaining tense or gaining a better understanding of the person with bvFTD, and becoming more confident in caring) and the results of the goal attainment appraisal are presented in Additional file 1: Table S1. The primary carers’ GAS ratings at t2 showed that 3 of 5 had reached their predefined goals more than expected, and 2 of 5 had reached them as expected.

### Pilot process analysis

#### Quantitative interviews and feedback questionnaires

The absolute frequencies of the responses of the primary carers from the quantitative interviews and written feedback questionnaires are presented in Figs. 3 and 4. Regarding the quantitative interviews, the participants agreed or completely agreed with 94% of the 27 items with positive polarity, and they did not agree with 91% of the 7 items with negative polarity (see Fig. 3).

A similar result was found for the quantitative feedback questionnaires. The participants agreed or completely agreed with 98% of the ratings of the eight positively poled items, and they did not agree with 97% of the three negatively poled items (see Fig. 4).

#### Qualitative semi-structured interviews

The following two main themes emerged in the five individual semi-structured interviews with the MM therapist, which were conducted one to 12 days after t2: (1.) “quality of the implementation of the intervention” consisting of the subthemes: “overall experience of the counselling”, “typical and special characteristics of the counselling”, “optimization potential”, “implementation” and “organization of the counselling” and (2.) “changes” consisting of the subthemes: “perceived changes”, “impact mechanisms”, “appropriate dose of the intervention” and “promoting and inhibiting factors”. In the following paragraphs, these themes are summarized (for a more detailed description, see Additional file 3: Table S3):

For the theme “quality of the implementation of the intervention”, the unique psychopathology of people with bvFTD (social cognition) required a special adaptation of the communication elements of MM, e.g. persons with bvFTD had to be communicated with on an emotional low level. The MM therapist got the impression that in comparison with Alzheimer’s dementia the persons with bvFTD want to be included more frequently in the counselling process. However, joint counselling was difficult because there was a tendency to talk about the person with bvFTD in his/her presence. Relatively frequent jointly conducted MM counselling sessions and the relatively frequent necessity to first discuss urgent themes, such as pre-death grief or pending changes in living situation, may be why the MM counselling sessions took, on average, approximately 15 min longer than usually (approximately 20 min). The organization of the MM counselling was unproblematic. The weekly counselling rhythm was interrupted only once.

For the theme “changes”, the MM therapist reported various changes, such as the person with bvFTD becoming overall more relaxed with a longer attention span; the dyad developing extended mutual contact time and intensity; the primary carer becoming calmer; and the primary carer initiating contact more consciously and emotionally with more social participation, decision-making opportunities and positive guidance. Raising awareness of the FSEs through video feedback was, as intended, the central impact mechanism of the counselling method. The primary carer’s acceptance of the illness of the person with bvFTD is a crucial promoting factor because the primary carer can only become truly involved in and benefit from the counselling if he/she has accepted the illness. Based on the experiences with counselling, the MM therapist recommended two follow-up sessions after five basic sessions for a sustainable effect of MM counselling. The MM therapist considered a 2-week interval for the sessions to be more favourable than a 1-week interval.

#### Research documentation

The results of the documentation of the recruitment and data collection processes are summarized as follows.

Regarding the recruitment process, health care providers, e.g. leaders of support groups for carers of people with dementia, dementia care network members and physicians, seemed interested in the project because interventions for people with bvFTD and their families are rare and the need is high. Even if a neurologist had diagnosed the person, confirmation of the diagnosis of bvFTD proved difficult since they did not necessarily apply current criteria [9]. In these cases, a reconstruction of the diagnostic process in a personal conversation with the diagnosing neurologist was necessary.

There was substantial doubt regarding whether the video feedback would be accepted by both the family and the person with bvFTD. However, the option to improve the quality of the relationship was the major reason for participation. The inclusion/exclusion criteria seemed to be adequate.

Data collection by telephone was feasible in most cases and took an average of 45 min. In contrast, performing the videography required the physical presence of the researcher on site, complex travel logistics, and a session time of up to 2 1/2 h (plus time for notes and return travel), making it far more demanding and time consuming. However, overall, conducting videography in the homecare setting was unproblematic. Despite the initial concerns and comments regarding video recordings during the first data session (t0), the participating dyads became more familiar with the procedure over time.

## Discussion

The aim of the study was to assess the feasibility of both administering MM counselling with people with bvFTD and their primary carers as well as to assess feasibility for conducting a future confirmatory trial to evaluate the effectiveness of this counselling method in this specific target group Therefore, we will first discuss the results of the pilot effect study and afterwards the result of the process evaluation.

One main result of the pilot effect analysis is that most of the outcomes and instruments used seem to be suitable to describe the effect of MM counselling in a future confirmatory trial. In particular, MM counselling showed clinically very significant effects on the positive affect of the persons with bvFTD and the primary carers’ stress experience due to BPSD. However, changes in the quality of the dyadic relationships, assessed only from primary carers, could also be described as clinically significant. Since an effect of MM counselling on the quality of the dyadic relationship is intended, this outcome may be a suitable primary outcome for a future confirmatory trial. However, for it to be a primary outcome, procedures should be developed to enhance the ability of people with bvFTD to complete the QCPR scale as much as possible.

Moreover, the GAS showed that primary carers achieved their personal goals as expected or even more than expected by means of MM counselling. However, the two instruments used to measure the interpersonal skills of the persons with bvFTD (the MM instrument) and the sensitivity of the primary carer (the SI) should be further developed or replaced by other instruments for a future confirmatory trial. For the SI, the coefficients for determining the interrater reliability did not reach a sufficient level for any of the 15 items, and for the MM instrument, the interrater reliability was sufficient for only two of the four items. Due to its developmental background, the SI focuses more on physical aspects of interactions in professional nursing care, such as physical access, safeguarding intimacy or physical touch. These interactions were less relevant for the dyads in this study, which may be the reason why the SI may not be suitable for assessing the sensitivity of primary carers in this target group. In contrast, the MM instrument seems to be better suited for assessing changes; however, the instruction of the instrument needs to be improved, and the training of the observer has to be intensified to achieve better interrater reliability.

The results of the process evaluation show that the acceptance of progressing dementia illness and the subsequent assumption of the carer role by a primary carer may be a central promoting factor for increasing the benefits of MM counselling. For instance, in session 2, three ratings of one of the primary carers was striking because the person completely agreed with the negatively poled items “I felt under pressure during the Marte Meo® counselling” and “I found the Marte Meo® advice today annoying” and did not agree with statement “The Marte Meo® counselling was good for me”. The MM therapist explained these ratings as reaction one of the primary carer to becoming conscious of her husband’s dependence on her in the counselling video for this session. This concern is directly interconnected with the issue of coping with ambiguous loss and pre-death or anticipatory grief [54] in dementia. That is, the person with dementia is still physically present but is increasingly mentally absent [55]. Primary carers avoiding openly expressing grief and not being able to accept, in the sense of acceptance and commitment therapy (ACT) and grief-associated negative feelings [56], could lead to complicated grief and depression. Moreover, primary carers can miss the opportunities to prepare themselves for the final loss of their partners with dementia, to adapt to their carer roles and to actively form the care process based on their values. Our results confirm that experiencing pre-death grief, with or without psychological support, may be a necessary prerequisite to benefitting from MM counselling.

Moreover pilot process evaluation showed that MM counselling worked with people with bvFTD and that MM counselling had very positive outcomes overall and in the individual counselling sessions that were evaluated, except for one session of one primary carer. However, the primary carers perceived the frequency of the counselling sessions as sufficient, whereas the MM therapist suggested additional follow-up sessions after a period of several weeks so that the counselling content can be implemented in daily life. Follow-up sessions have indeed been shown to be effective elements for all trainings and interventions in dementia care [57].

One new and unexpected aspect of the counselling process was that two people with bvFTD wanted to be included in the feedback process. Contrary to usual MM counselling, which is carried out mainly with the primary carer, and because all participating people with bvFTD had legal representatives, some of the people with bvFTD participated in watching the videos and getting feedback, even if their language abilities were very limited. This is especially remarkable because the perspectives of people with bvFTD are underrepresented and not well understood [58].

The recruitment process as a whole was very labourious and time consuming. The main motivation to take part in the study was the desire to improve the quality of the dyad relationship. Doubts about the benefits of video feedback and discomfort with videos exist and need to be addressed. Following current recommendations [33], we agree that barriers and anxieties can be addressed by providing more complete information, but even more through confidence-building initiatives by therapists and researchers during the whole procedure and emphasis of the positive factors of video feedback, e.g. learning by seeing oneself. The health care providers were convinced that the method would be useful. Thus, we assume that there would be substantial motivation on all sides (carers, persons with dementia and professionals) to support and participate in a confirmatory trial. A challenging aspect in the context of the recruitment of participants is the assurance of a valid bvFTD diagnosis. The review of the diagnoses in this study showed that even a bvFTD diagnosis made by a specialist (neurologist) is subject to uncertainty and that a diagnosis validated by a specialist is important.

Regarding the sample characteristics, some corresponds approximately to the general distribution like the mean age about 62 years of the person with bvFTD at disease onset. Although current studies show that men and women are nearly equally affected by the disease [4] and the majority of carers of people with FTLD are women [7], only female primary carers and one female person of bvFTD were willing to take part in the study. This is therefore important to look carefully at whether the finding would have been different in terms of the perceptions of male carers and more women with bvFTD.

### Limitations

The main limitation of our study is that we focused primarily on the feasibility of the intervention and less on the feasibility of a future confirmatory trial. For example, based on the available study results, we can make relatively few well-founded statements as to whether enough potential participants might consent to participate in a larger study, whether the target group might be able to be randomized, and what percent of the participants might be retained in a future study. Moreover, it might have been useful if the intervention process would have been investigated not only with qualitative interviews with the MM therapist but also with interviews with the primary carers. In our opinion, the view of the relatives would have been necessary to be able to understand the intervention process more fully.

### Recommendations for a future confirmatory trial

Finally, we want to provide some recommendation based on our findings for designing a future confirmatory trial:

- It is desirable to have an observation instrument available, which can directly assess interpersonal skills on a video basis and that does not rely on proxy ratings. In addition, the focus of MM counselling is on improving the communication skills of carers, and it could be useful to integrate the sense of competence questionnaire (SCQ) [59].
- Primary carers’ acceptance of the disease or at least an advanced mourning process regarding ambiguous loss in dementia seems to be crucial for the successful use of MM counselling and thus should be taken into account at least as a control variable, e.g. measured with the Caregiver Grief Scale [54], or even as an inclusion or exclusion criterion. Overall, the inclusion and exclusion criteria chosen for this study seem to be applicable for a confirmatory trial.
- The potential desire of people with bvFTD to participate must be taken into account for the administration of the intervention and the training of MM therapists in a future confirmatory trial and should definitely be supported and encouraged.
- For the following reasons, a large-scale definitive study should be performed as a multicentre study with specialized outpatient dementia clinics as the study centres. First, the likelihood of including (more) study participants with a confirmed diagnosis of bvFTD is higher if potential participants are recruited through specialized study centres. Second, a multicentre design seems useful in the context of including a large number of patients with rare conditions, such as bvFTD [60]. However, a multicentre design increases the effort, time and resources required for the study because more MM therapists are needed. There are currently approximately 700 trained and certified MM therapists in Germany [61], although very few of them have experience using the method with people with dementia. Specific training for MM therapists in counselling with people with dementia would have to be developed and implemented.
- Since this study showed that the recruitment of a larger sample of people with a diagnosis of bvFTD is very difficult and expensive and since nothing is known about evaluating MM counselling in a larger-scale, randomized trial (e.g. whether the target group could be randomized, the retention rate), a future confirmatory trial should be first planned as a pilot study with an extension option according to the confidence interval approach from Cocks and Torgerson [62]. Additionally, this study showed that telephone interviews were feasible for the selected outcomes and instruments. Therefore, in a possible subsequent multicentre confirmatory trial, data collection should also be conducted via telephone to save substantial financial (personal and travel costs) and time resources.

## Conclusions

The results provide indications of the feasibility of MM counselling with people with bvFTD and their primary carers in practise as well as of a large-scale confirmatory trial. With regard to the very limited treatment options and the low evidence of existing psychosocial interventions for this target group, further well-planned studies, following the listed recommendations for a video-based dyadic intervention, are of utmost importance.

## Supplementary information

Additional file 1: **Table S1.** Results of the Goal Attainment Scale (GAS).

Additional file 2: **Table S2.** German Version of the Quality of Carer-Patient Relationship (QCPR) Scale.

Additional file 3: **Table S3.** Summaries of the main themes and subthemes resulting from the qualitative structured content analysis of the interviews with the Marte Meo® therapist.

## Data Availability

The data sets used and analysed during the current study are available from the corresponding author on reasonable request.

## Abbreviations

AD: Alzheimer’s Disease
BPSD: Behavioural and Psychological Symptoms of Dementia
bvFTD: Behavioural variant frontotemporal dementia
DGP: Deutsche Gesellschaft für Pflegewissenschaft
FREQ: Frequency
FRS: Frontotemporal Dementia Rating Scale
FSEs: Function-Supporting Elements
FTLD: Frontotemporal Lobar Degeneration
GAS: Goal Attainment Scale
ICD-10: International Classification of Disease – Version 10
MAXQDA: MAX Qualitative Data Analysis
MM: Marte Meo®
MRC: Medical Research Council
NPI: Neuropsychiatric Inventory
QCPR: Quality of Carer-Patient Relationship
QoL: Quality of Life
RCT: Randomized Controlled Trial
SI: Sensitivity Index
TIDieR: Template for Intervention Description and Replication
VIA: Video Interaction Analysis
