# Supplementary material for: Application of Marte Meo® counselling with people with behavioural variant frontotemporal dementia and their primary carers (AMEO-FTD) – a non-randomized mixed-method feasibility study
Source: Pilot Feasibility Stud. 2020 Feb 26;6:32. doi: 10.1186/s40814-020-0551-1 (PMC7043032; doi:10.1186/s40814-020-0551-1)
Supplement: Supplementary file 1 — Table S1. Results of the Goal Attainment Scale (GAS). [file 40814_2020_551_MOESM1_ESM.pdf]

Additional file 1: **Table S1.** Results of the Goal Attainment Scale (GAS)

		**Case 1**	**Case 2**	**Case 3**	**Case 4**	**Case 5**
Goal Level*	Description*					
Much more than expected/much better success (+2)	-Complete attainment of goal criteria	Stress in the interaction was reduced substantially compared to the initial condition immediately after the counselling (50%).	The person with bvFTD is generally much more relaxed than before counselling and can be put to bed in the evening (+ 50%).	Positive moods in the interaction have become significantly more frequent and last much longer than before.	The carer understands much better and more often how her husband is, what he wants and what his true opinion is.	The carer is much more confident in caring for the person with bvFTD than before.
More than expected/better success (+1)	-Positive change from the initial state<br>-Partial attainment of goal criteria	Stress in the interaction was reduced a little compared to the initial condition immediately after the counselling (25%).	The person with bvFTD is somewhat more relaxed than before counselling but still lacks a day-night rhythm (+ 25%).	Positive moods in the interaction have become more frequent and last longer than before.	The carer understands better and more often how her husband is doing, what he wants and what his true opinion is.	The care is much more confident in caring for the person with bvFTD than before.
Expected success (0)	-Maintained the initial state<br>-Deterioration could be stopped	Stress in the interaction was maintained compared to the initial condition immediately after the counselling (± 0%).	The person with bvFTD is just as tense as before the consultations and still lacks a day-night rhythm (± 0%).	Positive moods in the interaction remain infrequent and last as long as before.	The carer understands as well and as often as ever how her husband is, what he wants and what his true opinion is.	The carer is as confident in caring for the person with bvFTD as before.
Less than expected/ worse success (+1)	-Partial or complete deterioration of defined goal criteria	Stress in the interaction increased slightly compared to the initial condition immediately after the counselling (25%).	The person with bvFTD is even more tense than before counselling and still lacks a day-night rhythm (-25%).	Positive moods in the interaction have become more infrequent and last shorter than before.	The carer understands worse and less frequently than before how her husband is, what he wants and what his true opinion is.	The carer is less confident in caring for the person with bvFTD than before.
Much less than expected/much worse success (+2)	-Complete deterioration of defined goal criteria	Stress in the interaction increased strongly compared to the initial condition immediately after the counselling (50%).	The person with bvFTD is much more tense than before counselling and still lacks a day-night rhythm (-25%).	Positive moods in the interaction have become much more infrequent and last much shorter than before.	The carer understands much worse and much less frequently than before how her husband is, what he wants and what his true opinion is.	The carer is less confident in caring with the person with bvFTD than before.

* The table cell of the description of the expected goal level is printed bold

bvFTD = behavioural variant frontotemporal dementia
