# Supplementary material for: Application of Marte Meo® counselling with people with behavioural variant frontotemporal dementia and their primary carers (AMEO-FTD) – a non-randomized mixed-method feasibility study
Source: Pilot Feasibility Stud. 2020 Feb 26;6:32. doi: 10.1186/s40814-020-0551-1 (PMC7043032; doi:10.1186/s40814-020-0551-1)
Supplement: Supplementary file 2 — Table S2. German Version of the Quality of Carer-Patient Relationship (QCPR) Scale. [file 40814_2020_551_MOESM2_ESM.docx]

Additional file 2: Table A2. German Version of the Quality of Carer-Patient Relationship (QCPR) Scale

| **Interviewer Instruktion:** Die Pflegeperson (Angehöriger) auf die sich hier bezogen wird, ist diejenige Person welche eine andere Person pflegt. Bitte die jeweilige Antwort durch ankreuzen der jeweiligen Antwortmöglichkeit für **jede** Reihe kenntlich machen.  **Verbale Instruktion für die befragte Person:** “Bitte denken Sie über Ihre Beziehung zu der Person nach, welche Sie betreuen bzw. pflegen und beantworten Sie die folgenden Fragen durch ankreuzen der jeweiligen Antwortmöglichkeit”  Antwortkategorien:  1 = Stimme überhaupt nicht zu  2 = Stimme nicht zu  3 = Nicht entschieden  4 = Stimme zu  5 = Stimme vollkommen zu | | | | | | |
| --- | --- | --- | --- | --- | --- | --- |
|  |  | Stimme überhaupt nicht zu | Stimme nicht zu | Nicht entschieden | Stimme zu | Stimme vollkommen zu |
| 1. | Wir verbringen oft auf eine angenehme Weise Zeit zusammen. | \| 1 \| \| --- \| | \| 2 \| \| --- \| | \| 3 \| \| --- \| | \| 4 \| \| --- \| | \| 5 \| \| --- \| |
| 2. | Wir sind oft unterschiedlicher Meinung. | \| 5 \| \| --- \| | \| 4 \| \| --- \| | \| 3 \| \| --- \| | \| 2 \| \| --- \| | \| 1 \| \| --- \| |
| 3. | Zwischen uns gibt es eine große Distanz. | \| 5 \| \| --- \| | \| 4 \| \| --- \| | \| 3 \| \| --- \| | \| 2 \| \| --- \| | \| 1 \| \| --- \| |
| 4. | Wir akzeptieren einander so, wie wir sind. | \| 1 \| \| --- \| | \| 2 \| \| --- \| | \| 3 \| \| --- \| | \| 4 \| \| --- \| | \| 5 \| \| --- \| |
| 5. | Wenn es Probleme gibt, können wir diese meistens schnell gemeinsam lösen. | \| 1 \| \| --- \| | \| 2 \| \| --- \| | \| 3 \| \| --- \| | \| 4 \| \| --- \| | \| 5 \| \| --- \| |
| 7. | Ich komme gut mit ihm/ihr aus. | \| 1 \| \| --- \| | \| 2 \| \| --- \| | \| 3 \| \| --- \| | \| 4 \| \| --- \| | \| 5 \| \| --- \| |
| 10. | Wir schenken einander Zärtlichkeit. | \| 1 \| \| --- \| | \| 2 \| \| --- \| | \| 3 \| \| --- \| | \| 4 \| \| --- \| | \| 5 \| \| --- \| |
| 11. | Ich ärgere mich häufig über die ihn/sie. | \| 5 \| \| --- \| | \| 4 \| \| --- \| | \| 3 \| \| --- \| | \| 2 \| \| --- \| | \| 1 \| \| --- \| |
| 13. | Wenn ich bei ihm/ihr bin, fühle ich mich sehr wohl. | \| 1 \| \| --- \| | \| 2 \| \| --- \| | \| 3 \| \| --- \| | \| 4 \| \| --- \| | \| 5 \| \| --- \| |
| 14. | Wir versuchen oft einander die eigene Meinung aufzudrängen. | \| 5 \| \| --- \| | \| 4 \| \| --- \| | \| 3 \| \| --- \| | \| 2 \| \| --- \| | \| 1 \| \| --- \| |
| 17. | Ich werfe ihm/ihr vor, die Ursache für meine Probleme zu sein. | \| 5 \| \| --- \| | \| 4 \| \| --- \| | \| 3 \| \| --- \| | \| 2 \| \| --- \| | \| 1 \| \| --- \| |
| 19. | Wir schätzen uns einander als Person. | \| 1 \| \| --- \| | \| 2 \| \| --- \| | \| 3 \| \| --- \| | \| 4 \| \| --- \| | \| 5 \| \| --- \| |
| 21 | Er/Sie schätzt nicht genügend, was alles ich für ihn/sie tue. | \| 5 \| \| --- \| | \| 4 \| \| --- \| | \| 3 \| \| --- \| | \| 2 \| \| --- \| | \| 1 \| \| --- \| |
| 22. | Wenn ich ihn/sie eine Weile nicht gesehen habe, freue ich mich ihn/sie wieder zu sehen. | \| 1 \| \| --- \| | \| 2 \| \| --- \| | \| 3 \| \| --- \| | \| 4 \| \| --- \| | \| 5 \| \| --- \| |
| 'Positive' Subskala „Wärme und Zuneigung“:  Items 1, 4, 5, 7, 10, 13, 19, 22  'Negative' Subskala „Konflikt und Kritik“  Items 2, 3, 11, 14, 17, 21 (diese Items werden umgekehrt gewertet) | | | | | | |
